# Current Practices and Trends of Plastic and Oncoplastic Breast Surgeons in Canada

**DOI:** 10.1177/22925503231195020

**Published:** 2023-08-21

**Authors:** Brendon Bitoiu, Emma Grigor, Camille Zeitouni, Angel Arnaout, Jing Zhang

**Affiliations:** 1 Department of Surgery, Division of Plastics and Reconstructive Surgery, The Ottawa Hospital, Ottawa, ON, Canada; 2 MD Program, Faculty of Medicine, 6363 University of Ottawa, Ottawa, ON, Canada; 3 Clinical Epidemiology Program, Ottawa Hospital Research Institute, Ottawa, ON, Canada; 4 Department of Surgery, Division of General Surgery, The Ottawa Hospital, Ottawa, ON, Canada

**Keywords:** survey, breast reconstruction, oncoplastic surgery, chirurgie oncoplastique, reconstruction mammaire, sein

## Abstract

**Background:** There is a lack of previous studies investigating oncoplastic practice trends for breast reconstruction in Canada, particularly from the plastic surgeon perspective. Given the rising popularity of oncoplastic techniques, this study aimed to identify current practice trends for breast and plastic surgeons in Canada. **Methods:** A cross-sectional survey study of breast and plastic surgeons performing oncoplastic surgery across Canada was conducted. **Results:** Ninety-five surgeons were invited to complete the survey, with 58 respondents (response rate 61%), of which 29 (50.0%) were breast surgeons and 29 (50.0%) were plastic surgeons. Compared to plastic surgeons, breast surgeons performed significantly more level 1 surgeries (27.6 vs 3.45%, *P* < .001). Plastic surgeons performed more level 2 (37.9% vs 13.8%, *P* = .0475) and level 3 (31.4% vs 10.3%, *P* = .00814) surgeries. Breast surgeons identified significant perceived barriers including unfamiliarity with techniques (*P* = .00513), adjuvant therapy delays (*P* = .00612), lack of plastic surgery support (*P* < .001), lack of radiation oncology support (*P* = .0485), increased OR time (*P* < .001), lack of OHIP billing codes (*P* < .001), and post-operative complication management (*P* = .0372). Breast surgeon comfort with oncoplastic techniques was not correlated with practice duration (R-square = .037, *P*-value = .853). Breast surgeon comfort with contralateral surgery was not correlated with practice setting (R-square = .071, *P*-value = .632). **Conclusions:** Breast surgeons perceive a lack of training, a lack of support from plastic surgery, concerns regarding appropriate financial remuneration, and worries of increased OR time as barriers in oncoplastic surgery. Collaboration between general breast surgery and plastic surgery is needed for improving training options for oncoplastic surgery in Canada and for providing excellent breast cancer care overall.

## Résumé

**Historique :** Peu d’études ont porté sur les tendances des chirurgies mammaires en oncoplastie au Canada, particulièrement selon le point de vue des plasticiens. Étant donné la popularité croissante des techniques d’oncoplastie, la présente étude visait à déterminer les tendances chez les chirurgiens du sein et les plasticiens du Canada. **Méthodologie :** Les chercheurs ont réalisé une étude transversale auprès des chirurgiens du sein et des plasticiens qui effectuent des chirurgies oncoplastiques au Canada. **Résultats :** Au total, 95 chirurgiens ont été invités à remplir le sondage et 58 ont répondu (taux de réponse de 61%), soit 29 chirurgiens du sein (50,0%) et 29 plasticiens (50,0%). Les chirurgiens du sein effectuaient beaucoup plus de chirurgies de niveau 1 que les plasticiens (27,6% par rapport à 3,45%, *P* < ,001). Les plasticiens effectuaient plus de chirurgies de niveau 2 (37,9% par rapport à 13,8%, *P* = ,0475) et de niveau 3 (31,4% par rapport à 10,3%, *P* = ,00814). Les chirurgiens du sein ont souligné percevoir d’importants obstacles, y compris la méconnaissance des techniques (*P* = ,00513), l’attente avant d’obtenir des traitements adjuvants (*P* = ,00612), l’absence de soutien en chirurgie plastique (*P* < ,001) et en radio-oncologie (*P* = ,0485), le temps accru en salle d’opération (*P* < ,001), l’absence de codes de facturation du RAMO (*P* < ,001) et la prise en charge des complications postopératoires (*P* = ,0372). **Conclusions :** Les chirurgiens perçoivent l’absence de formation, l’absence de soutien de la chirurgie plastique, la crainte de ne pas recevoir de rémunération appropriée et la possibilité d’accroître leur temps en salle d’opération comme des obstacles à la chirurgie oncoplastique. Une collaboration entre la chirurgie mammaire générale et la chirurgie plastique s’impose pour améliorer les possibilités de formation en oncoplastie au Canada et prodiguer d’excellents soins globaux en cas de cancer du sein.

## Introduction

The popularization of oncoplastic surgery has increased in recent years with the advancement in surgical techniques.^1^ First described by Audretsch et al and Clough et al, oncoplastic breast conserving surgery involves the recreation of the breast mound following partial mastectomy and tumor resection.^2,3^ Similar oncologic outcomes have been shown in patients treated with breast-conserving procedure followed by radiation therapy, and those opting for mastectomy with reconstruction.^4^ Despite the widespread acceptance and prevalence of oncoplastic breast surgery in European countries, North America has had a more delayed adoption of this technique.^5^ A recent Canadian study reported unfamiliarity with the technique, lack of training opportunities, and lack of support from plastic surgery to be the main reasons breast surgeons hesitated to utilize oncoplastic techniques among general surgeons.^6^ More so, it remains unclear at which point the plastic surgeon is indicated for involvement. Clough et al has suggested level 1 training may not require the involvement of a plastic surgeon or additional training, whereas level 2, and more advanced oncoplastic techniques require additional training and/or plastic surgery involvement.^6^ Although plastic surgeons have historically been responsible for breast reduction surgery and tissue transfer reconstruction, with the increase in focused fellowships for breast surgeons, this may change in the future.^5^ A collaborative approach between the plastic surgeon and breast surgeon may eventually popularize as a conventional approach in treating breast cancer.

To date, there is no study done exploring current practice trends among both breast surgery and plastic surgery communities in Canada. Previous studies surveying oncoplastic breast conserving surgery trends and guidelines in Canada have involved a general surgery perspective.^5^ Given the rise in oncoplastic surgery and the variability that currently exists among surgeons offering these procedures, this study aims to identify current practice trends for both plastic surgeons and oncoplastic breast surgeons in Canada.

## Methods

We performed a cross-sectional survey study of Canadian breast and plastic surgeons who were identified as providing oncoplastic surgery at their practice. Research ethics board approval (Protocol ID: 20220043-H1) was granted for a prospective observational survey study between February 2022 and June 2022. Registered Canadian plastic surgeons and general surgeons specializing in breast surgery were considered eligible to participate. Surgeons with subspecialty practice in vascular, thoracic, and pediatric surgery were excluded. This study adhered to the STROBE guideline for reporting observational studies (Supplement 1).^7^

Both breast and plastic surgeons were identified through central emailing lists, respectively. Eligible participants were emailed a link to access the oncoplastic survey over email and data was collected online using SurveyMonkey. The questionnaire was developed based on current literature regarding oncoplastic surgery in practice.^6^ The survey consisted of a 11-item questionnaire to collect surgeon and practice demographics, use of oncoplastic techniques, and barriers to oncoplastic use in practice. Surgeon and practice demographics included time in practice (years), type of practice (academic hospital vs community center), number of oncoplastic patients per year, types of oncoplastic techniques integrated, level of surgery offered, percentage of practice involving breast surgery or plastic surgery, laterality of surgery, contralateral balancing procedures, and reasons against using oncoplastic surgery technique (Supplement 2). Reasons against using oncoplastic surgery techniques included lack of training, concerns regarding delay of adjuvant treatment, management of positive margins, poor cosmesis, lack of access to plastic surgeons and/or radiation oncology, patient interest, increased operative time, and lack of applicable billing codes. Respondents were also provided the option to qualitatively detail barriers to oncoplastic technique integration from their perspective.

### Statistical Analysis

The responses of breast surgeons and plastic surgeons were compared. The Mann–Whitney *U*-test was used to compare continuous data and chi-square (or Fisher exact) test for categorical data. Missing values were included as a separate category. Linear regression was performed to determine whether breast surgeon comfort with oncoplastic procedures was explained by practice duration (number of years) or practice type (academic vs community). Effect estimates and R-square statistics were obtained to assess for any possible correlation. R-square values (range from 0 to 1) that were closer to zero (0) meant little correlation whereas values closer to one (1) meant greater correlation. *P*-values < .05 were taken as statistically significant. The statistical analysis was performed using R studio (open source, version 1.3).

## Results

Of 95 Canadian surgeons contacted, there were 48 breast surgeons (50.5%) and 47 plastic surgeons (49.5%) invited to participate in the study. There were 58 respondents to the survey with a response rate of 61% (58 out of 95), of which 29 (50.0%) were breast surgeons and 29 (50.0%) were plastic surgeons.

Surgeon practice and oncoplastic technique characteristics are summarized according to breast and plastic surgeons in Table 1. The majority of surgeons were in Ontario (75.9%) with other respondents located in Quebec (8.62%), British Columbia (6.90%), Alberta (6.90%), and Manitoba (1.72%). There were significantly more breast surgeons in community practice compared to plastic surgeons (41.4% vs 17.2%), and fewer breast surgeons in academic practice compared to plastic surgeons (41.4% vs 79.3%) (*P* = .00631). Breast and plastic surgeon groups were similar in terms of years in practice. Breast surgeons reported performing significantly more level 1 displacement surgeries compared to plastic surgeons (27.6 vs 3.45%, *P* < .001). Plastic surgeons reported performing significantly more level 2 displacement (37.9% vs 13.8%, *P* = .0475) and level 3 replacement (31.4% vs 10.3%, *P* = .00814) surgery compared to breast surgeons. Breast surgeons, compared to plastic surgeons, utilized significantly more circumareolar Benelli mastopexy (75.9% vs 48.3%, *P* = .0482) and crescent mastopexy (79.3% vs 24.1%, *P* < .001) techniques. Plastic surgeons, compared to breast surgeons, utilized significantly more vertical scar mammaplasty (82.8% vs 44.8%, *P* = .00629). Flap techniques were primarily performed by plastic surgeons with glandular flaps being the most common type (72.4%), latissimus dorsi (LD) myocutaneous flaps (55.2%), thoracodorsal artery perforator (TDAP) flaps (44.8%), and LD muscle flaps (41.4%). Other flaps utilized by plastic surgeons included intercostal artery perforator flaps (27.6%), pedicled transverse rectus abdominis muscle flaps (20.7%), and superior epigastric artery perforator flaps (13.8%). Contralateral balancing procedures were performed in 100% of plastic surgeons and 48.3% of breast surgeons (*P* < .001). The majority of plastic surgeons felt that plastic surgeons should perform the contralateral balancing procedures compared to breast surgeons (96.6% vs 3.45%, *P* < .001), whereas breast surgeons felt either plastic or breast surgeons should perform contralateral balancing procedures (48.3% vs 44.8%, *P* < .001). Breast surgeons felt that level 2 and 3 displacement surgeries required referral level for a plastic surgeon (83.0%), whereas plastic surgeons felt that levels 1 to 3 all required referral for a plastic surgeon (93.1%) (*P* < .001). Plastic surgeons reported that the majority of their oncoplastic surgeries were referrals from breast surgeons (86.2%).

**Table 1.** Surgeon Characteristics and Oncoplastic Techniques (Breast Surgeon vs Plastic Surgeon).

	Total N = 58	Breast n = 29	Plastic n = 29	*P*-value
Province, no. (%)				
Ontario	44 (75.9)	25 (86.2)	19 (65.5)	
Quebec	5 (8.62)	2 (6.90)	3 (10.3)	
British Columbia	4 (6.90)	0 (0)	4 (13.8)	.0747
Alberta	4 (6.90)	2 (6.90)	2 (6.90)	
Manitoba	1 (1.72)	0 (0)	1 (3.45)	
Practice type, no. (%)				
Academic	35 (60.3)	12 (41.4)	23 (79.3)	
Community	17 (29.3)	12 (41.4)	5 (17.2)	.00631*
Combined	6 (10.3)	5 (17.2)	1 (3.45)	
Practice duration, no. (%)				
<5 years	17 (29.3)	7 (24.1)	10 (17.0)	
5-10 years	13 (22.4)	6 (20.7)	7 (24.1)	
11-15 years	13 (22.4)	7 (24.1)	5 (17.2)	.748
16-20 years	9 (31.0)	6 (20.7)	3 (10.3)	
>20 years	7 (12.1)	3 (10.3)	4 (13.8)	
Patient no. per year, mean (±SD)	15.8 (11.7)	12.8 (5.15)	18.9 (5.25)	.657
Plastic surgeon use (%), mean (±SD)	67.8 (36.8)	31.9 (19.8)	100 (0)	<.001*
Techniques used, no. (%)				
Circumareolar Benelli mastopexy	36 (62.1)	22 (75.9)	14 (48.3)	.0482*
Vertical scar mammaplasty	37 (63.8)	13 (44.8)	24 (82.8)	.00629*
Crescent mastopexy	30 (51.7)	23 (79.3)	7 (24.1)	<.001*
Glandular flaps	21 (36.2)	0 (0)	21 (72.4)	<.001*
LD myocutaneous flaps	17 (29.3)	1 (3.45)	16 (55.2)	<.001*
TDAP flaps	14 (24.1)	1 (3.45)	13 (44.8)	.000737*
LD muscle flaps	12 (20.7)	0 (0)	12 (41.4)	.000363*
ICAP flaps	9 (15.5)	1 (3.45)	8 (27.6)	.0296*
Pedicled TRAM flaps	6 (10.3)	0 (0)	6 (20.7)	.0311*
SEAP flaps	5 (8.62)	1 (3.45)	4 (13.8)	.349
Level 1 volume displaced, no. (%)				
≤50%	42 (72.4)	15 (51.7)	27 (93.1)	
>50%	14 (24.1)	13 (27.6)	1 (3.45)	<.001*
*Not reported*	1 (1.72)	0 (0)	1 (3.45)	
Level 2 volume displaced, no. (%)				
≤50%	41 (70.7)	24 (82.8)	17 (58.6)	
>50%	15 (25.9)	4 (13.8)	11 (37.9)	.0475*
*Not reported*	1 (1.72)	0 (0)	1 (3.45)	
Level 3 volume replacement, no. (%)				
≤50%	39 (67.2)	22 (75.9)	17 (58.6)	
>50%	15 (25.9)	3 (10.3)	12 (41.4)	.00814*
*Not reported*	3 (5.17)	3 (10.3)	0 (0)	
Contralateral surgery performed, no. (%)				
Yes	43 (74.1)	14 (48.3)	29 (100)	<.001*
No	14 (24.1)	14 (48.3)	0 (0)	
Contralateral surgeon type, no. (%)				
Breast surgeon	14 (24.1)	13 (44.8)	1 (3.45)	<.001*
Plastic surgeon	42 (72.4)	14 (48.3)	28 (96.6)	
Referral level for plastic surgeon, no. (%)				
Level 1	29 (50.0)	28 (83.0)	1 (3.45)	
Level 2	1 (1.72)	0 (0)	1 (3.45)	<.001*
Level 3	0 (0)	0 (0)	0 (0)	
Levels 1-3	27 (46.6)	0 (0)	27 (93.1)	
Referrals from breast surgeons, no. (%)				
<25%	2 (3.45)	0 (0)	2 (6.90)	
25%-50%	1 (1.72)	0 (0)	1 (3.45)	1.00
50%-75%	4 (6.90)	0 (0)	4 (13.8)	
>75%	21 (36.2)	0 (0)	21 (72.4)	

Abbreviations: ICAP, inter-costal artery perforator; LD, latissimus dorsi; no., number; N/A, not applicable; SD, standard deviation; SEAP, superior epigastric artery perforator; TDAP, thoracodorsal artery perforator; TRAM, transverse rectus abdominis muscle.

*P-value < .05 statistically significant.

Surgeon reported reasons against using oncoplastic surgery techniques are summarized according to breast and plastic surgeons in Table 2. Breast surgeons identified the following as significant barriers to the use of oncoplastic surgery techniques, whereas plastic surgeons did not: unfamiliarity with techniques (2.18 ± 1.42 vs 1.28 ± 0.800, *P* = .00513), delays to adjuvant therapy (2.00 ± 1.30 vs 1.69 ± 0.806, *P* = .00612), lack of support from plastic surgery (2.39 ± 1.9 vs 0.966 ± 0.731, *P* < .001), lack of support from radiation oncology (2.00 ± 1.33 vs 1.38 ± 0.942, *P* = .0485), increased OR time (2.54 ± 1.20 vs 1.66 ± 0.936, *P* < .001), lack of OHIP billing codes (2.82 ± 0.983 vs 1.93 ± 0.998, *P* < .001), and management of post-operative complications (2.32 ± 1.36 vs 1.69 ± 0.761, *P* = .0372). Tables 3 and 4 provide insights into the factors influencing breast surgeon comfort with oncoplastic techniques and performing the contralateral procedure. The analysis reveals that practice duration did not significantly explain breast surgeon comfort with oncoplastic techniques (R-square = .037, *P*-value = .853) (Table 3). Similarly, practice setting did not account for the comfort level of breast surgeons in performing the contralateral procedure (R-square = .071, *P*-value = .632) (Table 4).

**Table 2.** Surgeon Reasons Against Oncoplastic Surgery Techniques (Breast Surgeon vs Plastic Surgeon).

	Total N = 58	Breast n = 29	Plastic n = 29	
Reasons against oncoplastic surgery techniques (0-4)	Mean (SD)	Mean (SD)	Mean (SD)	*P*-value
I am unfamiliar with these techniques	1.72 (1.22)	2.18 (1.42)	1.28 (0.800)	.00513*
I am concerned about delay of adjuvant treatment	1.84 (1.08)	2.00 (1.30)	1.69 (0.806)	<.00612*
I am concerned about the need for re-operation for positive margins	2.18 (1.10)	2.00 (1.33)	2.34 (0.814)	.247
I do not have support from plastic surgery	1.67 (1.31)	2.39 (1.39)	0.966 (0.731)	<.001*
I do not have support from radiation oncology	1.68 (1.18)	2.00 (1.33)	1.38 (0.942)	.0485*
I am concerned about increased OR time	2.09 (1.15)	2.54 (1.20)	1.66 (0.936)	<.001*
I am concerned about the lack of specific OHIP billing codes	2.37 (1.08)	2.82 (0.983)	1.93 (0.998)	<.001*
I am concerned about poor cosmesis	1.89 (1.08)	2.18 (1.28)	1.62 (0.915)	.0535
My patients are not interested	2.00 (1.07)	2.14 (1.21)	1.86 (0.731)	.234
I am concerned about the rate of post-operative complications	2.02 (1.04)	2.25 (1.26)	1.79 (0.726)	.103
I am concerned about managing post-operative complications	2.00 (1.13)	2.32 (1.36)	1.69 (0.761)	.0372*
Reasons against oncoplastic surgery techniques: option for surgeons to provide detailed responses *Respondent 1 [plastic surgeon]*: “A strong concern is possibility of positive margins and need for completion mastectomy” *Respondent 2 [plastic surgeon]*: “An important reason is the potential for complications and future revision surgery, which highlights the need for a plastic surgeon as the primary surgeon for oncoplastic surgery methods” *Respondent 3 [breast surgeon]*: “I've had patients who were candidates for partial breast irradiation who would no longer be eligible for partial breast irradiation if they received oncoplastic techniques, which would necessitate whole breast irradiation. In these situations, I opt for a conventional lumpectomy with closure of the lumpectomy cavity. If there are issues with cosmesis following radiation, I refer patients to a plastic surgeon”				
Survey response scale 0 N/A 1 Strongly Disagree 2 Disagree 3 Agree 4 Strongly Agree				

Abbreviations: N/A, not applicable; SD, standard deviation.

*P-value < .05 statistically significant.

**Table 3.** Breast Surgeon Comfort With Oncoplastic Surgery Techniques and Practice Duration.

Variable	Estimate	SE	R-square**	*P*-value*
Practice duration	−0.0302	0.161	.037	.853

Abbreviations: CI, confidence interval; N/A, not applicable; SE, standard error.

*Statistically significant for *P* ≤ .05% and 95% CI not crossing null value (1.0).

**R-square statistic: *R* = 0 means surgeon comfort is not related to practice duration and *R* = 1 means surgeon comfort is perfectly explained by practice duration.

**Table 4.** Breast Surgeon Comfort With Contralateral Procedure and Practice Setting.

Variable	Estimate	SE	R-square**	*P*-value*
Practice duration				
Community	*Reference*	–	–	–
Academic	0.0345	0.161	.071	.632

Abbreviations: CI, confidence interval; N/A, not applicable; SE, standard error.

*Statistically significant for *P* ≤ .05% and 95% CI not crossing null value (1.0).

**R-square statistic: *R* = 0 means surgeon comfort is not related to the practice setting (academic vs community) and *R* = 1 means surgeon comfort is perfectly explained by practice setting.

## Discussion

This prospective survey study was the first study to examine both breast surgery and plastic surgeon perspective regarding oncoplastic surgery in Canada. We found that most level 1 displacement surgeries were performed by breast surgeons while plastic surgeons performed the majority of level 2 and level 3 displacement surgeries. All plastic surgeons reported performing contralateral balancing procedures and the majority believed that these procedures should be performed by a plastic surgeon. Less than half of respondent breast surgeons performed contralateral balancing procedures and considered either breast or plastic surgeons as appropriate to perform this procedure. Interestingly, neither time in practice nor practice setting significantly affected breast surgeon comfort level. Lastly, breast surgeons reported unfamiliarity with oncoplastic surgery techniques, concerns about delay for adjuvant treatment, managing post-operative complications, not having support from plastic surgery or radiation oncology, concerns about the increased OR time, along with a lack of specific billing codes as barriers in performing oncoplastic breast surgery, while plastic surgeons did not.

Oncoplastic techniques are classified into various levels determined by the complexity and advanced skill required for the procedure. It is suggested that level 1 oncoplastic surgery does not require additional training in breast surgery.^6,8^ This is consistent with our survey findings as most level 1 displacement surgeries were performed by breast surgeons. Conversely, level 2 and level 3 oncoplastic surgery requires additional training either through formal oncoplastic fellowship training or oncoplastic hands on courses, or plastic surgery training.^6,8^ This parallels our findings as most level 2 and level 3 displacement surgeries were performed by plastic surgeons.

A lack of training was found to be a barrier for breast surgeons in adopting oncoplastic surgery in their practices, however this was not the case for plastic surgeons. This finding is consistent with other studies exploring the barriers for oncoplastic surgery adoption for breast surgeons within clinical practice.^6,9–12^ In reality, most practicing breast surgeons in North America who currently perform oncoplastic surgery have developed their expertise through personal experience and courses done outside their formal training.^8–10^ In a 2017 American Society of Breast Surgeon Survey, 63.6% respondents indicated acquiring their skills using courses and 43.5% reported partnering with a plastic surgeon to acquire oncoplastic surgery skills.^10^ This highlights that factors such as individual learning experiences, exposure to oncoplastic techniques outside formal training, and ongoing professional development opportunities are what account for variation in comfort level, and why length of time in practice alone simply doesn’t account for that difference. The lack of formal training in oncoplastic surgery for breast surgeons lags the international community where breast surgeons are formally trained in oncologic and reconstructive principles through oncoplastic training programs.^6,9,10,13^ It may be then reasonable to believe that with greater integration of oncoplastic breast surgery techniques into general surgery training, breast surgeons can achieve similar levels of competence in oncoplastic surgery without the need to seek additional training elsewhere.^5,6,8,10^

Breast surgeons identified a lack of access and support from plastic surgery as a barrier for oncoplastic surgery integration in their practice. However, comparable comfort for techniques such as balancing procedures were found for breast surgeons in academic and community practice, seemingly contradicting the notion that access to plastic surgeons significantly impacts breast surgeons’ comfort with higher level oncoplastic techniques. Plastic surgeons also did not perceive this as a barrier. Lack of access and support parallels a previous study that surveyed Ontario breast surgeons regarding barriers for oncoplastic surgery integration in their practice.^6^ According to that survey, breast surgeons were less likely to perform oncoplastic surgery when they did not have access to plastic surgery, highlighting that plastic surgeons still have a major role in supporting oncoplastic surgery in Canada.^6^

Additionally, concern about increased OR time was one barrier identified by breast surgeons in our study that was not reported by plastic surgeons. This finding was inconsistent from a previous survey of barriers in oncoplastic integration by breast surgeons in Ontario where OR time was not identified as a barrier.^6^ An explanation for this may stem from the COVID-19 pandemic, as OR access during this time was severely limited, including access for breast reconstruction.^14,15^ This, along with ongoing staffing shortages have created immense backlogs in surgical care, making OR time a scarce resource.^14,15^ Additionally, breast surgeons in community practice do not have the same resources as surgeons in tertiary and academic settings, which is where most plastic surgeons are located.^16^ In this context, oncoplastic surgery may make even more sense within a resource limited setting as patients receiving oncoplastic surgery have fewer complications, compared to mastectomy and IBR, and less burden on hospital resources with shorter operative times and outpatient follow up.^15^ Operative time for oncoplastic surgery has also shown to gradually shorten as surgeons gain experience and develop “mastery” of the techniques.^17^ Nevertheless, an increase allocation of resources in performing oncoplastic surgery within community practice is needed for providing access to oncoplastic surgery to patients to both the breast and plastic surgeon alike.

Addressing the barriers to oncoplastic surgery noted by breast surgeons requires a multifaceted approach. One approach to improving oncoplastic surgery is through comprehensive training and education. This includes formal oncoplastic fellowships as well as practical professional development courses. The Canadian Oncoplastic Partnership Group, composed of experienced academic and community breast surgeons, offers full-day hands-on courses in Canada. Their objective is to enhance the quality of breast surgery. Results indicate that more than 90% of participating surgeons reported an increased utilization of oncoplastic surgery techniques after attending the 1-day course.^8–10^ A second solution involves fostering greater collaboration between breast and plastic surgeons which has proven successful in establishing formal oncoplastic fellowships co-direct by both specialties.^18^ To facilitate access for community surgeons, the establishment of referral networks can ensure timely consultations with oncoplastic or plastic surgeons.^18^ Overcoming the barrier of limited operating room time for oncoplastic surgery by breast surgeons necessitates advocating for increased OR resources at the hospital and provincial levels for breast cancer care. Additionally, it requires acknowledgment from breast surgeons and clinical care coordinators that operative times will decrease as surgeons gain expertise in oncoplastic techniques.^17^

Our study had several limitations including using an adapted survey used to poll breast surgeons on their perceptions of the oncoplastic landscape and barriers for integration in clinical practice.^6^ This survey has not been validated, and therefore its predictive power is unclear. Moreover, our response rate was 61%, this along with the nature of survey research, poses a limitation of selection bias within our results.

## Conclusion

In conclusion, our results contribute to continued understanding of the increasing oncoplastic surgery landscape across Canada by identifying barriers to work on while also highlighting the role plastic surgeons have in supporting oncoplastic surgery. Our results demonstrate that breast surgeons perceive a lack of training, a lack of support from plastic surgery, concerns regarding appropriate financial remuneration, and worries of increased OR time as these barriers. Collaboration between general breast surgery and plastic surgery is needed for improving training options for oncoplastic surgery in Canada and for providing excellent breast cancer care overall.

## Supplemental Material

sj-docx-1-psg-10.1177_22925503231195020 - Supplemental material for Current Practices and Trends of Plastic and Oncoplastic Breast Surgeons in Canada Supplemental material, sj-docx-1-psg-10.1177_22925503231195020 for Current Practices and Trends of Plastic and Oncoplastic Breast Surgeons in Canada by Brendon Bitoiu, Emma Grigor, Camille Zeitouni, Angel Arnaout and Jing Zhang in Plastic Surgery
