# Supplementary material for: Current Practices and Trends of Plastic and Oncoplastic Breast Surgeons in Canada
Source: Plast Surg (Oakv). 2023 Aug 21;33(1):35–41. doi: 10.1177/22925503231195020 (PMC11770734; doi:10.1177/22925503231195020)
Supplement: sj-docx-1-psg-10.1177_22925503231195020 - Supplemental material for Current Practices and Trends of Plastic and Oncoplastic Breast Surgeons in Canada [file sj-docx-1-psg-10.1177_22925503231195020.docx]

**Supplement 1.** STROBE guideline for reporting observational studies

| **Criteria** | **Page no.** | **Recommendation** |
| --- | --- | --- |
| **Title and abstract** | 1-2 | (*a*) Indicate the study’s design with a commonly used term in the title or the abstract |
|  |  | (*b*) Provide in the abstract an informative and balanced summary of what was done and what was found |
| **Introduction** | | |
| Background/rationale | 3 | Explain the scientific background and rationale for the investigation being reported |
| Objectives | 3 | State specific objectives, including any prespecified hypotheses |
| **Methods** | | |
| Study design | 4-5 | Present key elements of study design early in the paper |
| Setting | 4 | Describe the setting, locations, and relevant dates, including periods of recruitment, exposure, follow-up, and data collection |
| Participants | 4 | (*a*) Give the eligibility criteria, and the sources and methods of selection of participants. Describe methods of follow-up |
|  |  | (*b*) For matched studies, give matching criteria and number of exposed and unexposed |
| Variables | 4-5 | Clearly define all outcomes, exposures, predictors, potential confounders, and effect modifiers. Give diagnostic criteria, if applicable |
| Data sources/ measurement | 4-5 | For each variable of interest, give sources of data and details of methods of assessment (measurement). Describe comparability of assessment methods if there is more than one group |
| Bias | 4-5 | Describe any efforts to address potential sources of bias |
| Study size | NA | Explain how the study size was arrived at |
| Quantitative variables | 4-5 | Explain how quantitative variables were handled in the analyses. If applicable, describe which groupings were chosen and why |
| Statistical methods | 5 | (*a*) Describe all statistical methods, including those used to control for confounding |
|  |  | (*b*) Describe any methods used to examine subgroups and interactions |
|  |  | (*c*) Explain how missing data were addressed |
|  |  | (*d*) If applicable, explain how loss to follow-up was addressed |
|  |  | (*e*) Describe any sensitivity analyses |
| **Results** | | |
| Participants | 5-6 | (a) Report numbers of individuals at each stage of study—e.g. numbers potentially eligible, examined for eligibility, confirmed eligible, included in the study, completing follow-up, and analysed |
|  |  | (b) Give reasons for non-participation at each stage |
|  |  | (c) Consider use of a flow diagram |
| Descriptive data | 5-6 | (a) Give characteristics of study participants (e.g. demographic, clinical, social) and information on exposures and potential confounders |
|  |  | (b) Indicate number of participants with missing data for each variable of interest |
|  |  | (c) Summarise follow-up time (e.g., average, and total amount) |
| Outcome data | 5-6 | Report numbers of outcome events or summary measures over time |
| Main results | 5-6 | (*a*) Give unadjusted estimates and, if applicable, confounder-adjusted estimates and their precision (e.g., 95% confidence interval). Make clear which confounders were adjusted for and why they were included |
|  |  | (*b*) Report category boundaries when continuous variables were categorized |
|  |  | (*c*) If relevant, consider translating estimates of relative risk into absolute risk for a meaningful time period |
| **Discussion** | | |
| Key results | 7-8 | Summarise key results with reference to study objectives |
| Limitations | 9 | Discuss limitations of the study, taking into account sources of potential bias or imprecision. Discuss both direction and magnitude of any potential bias |
| Interpretation | 7-9 | Give a cautious overall interpretation of results considering objectives, limitations, multiplicity of analyses, results from similar studies, and other relevant evidence |
| Generalisability | 7-9 | Discuss the generalisability (external validity) of the study results |
| **Other information** | | |
| Funding | 1 | Give the source of funding and the role of the funders for the present study and, if applicable, for the original study on which the present article is based |

**Supplement 2.** Oncoplastic Survey Questionnaire

1. How many years have you been in practice?
   1. <5 years
   2. 5 – 10 years
   3. 11 – 15 years
   4. 16 – 20 years
   5. >21 years
2. What type of hospital do you work in?
   1. Academic
   2. Community
   3. Regional cancer center
   4. Other, please specify:
3. If you are a general surgeon, do you have subspecialty fellowship training in breast surgery, surgical oncology or plastic and reconstructive surgery?^3,7,8^
   1. If yes, please indicate which type of fellowship:
      1. Breast surgery
      2. Surgical oncology
      3. Plastic and reconstructive surgery
4. How many patients per year do you perform oncoplastic surgery on?
5. Which of the following techniques do you use when performing oncoplastic surgery?
   1. Skin incision planned for optimal cosmesis
   2. Undermining of skin
   3. Undermining of nipple areolar complex
   4. Leave lumpectomy cavity open and allow for seroma formation
   5. Glandular flaps
   6. Crescent mastopexy
   7. Circumareolar/Benelli mastopexy
   8. Batwing Mastopexy
   9. Clamshell technique
   10. Central excision and inverted-T mastopexy
   11. Vertical scar mammaplasty
   12. Wise pattern mammaplasty
6. Based on this classification system, what percentage would you say you perform in each of these levels (total of 100% amongst all 3 categories)?
   1. Volume displacement level 1 (<20% of breast tissue excised and closing the lumpectomy defect with redistribution of existing breast tissue)
      1. 0-25%
      2. 25-50%
      3. 50-75%
      4. 75-100%
   2. Volume displacement level 2 (20-50% of breast tissue excised and closing the lumpectomy defect with redistribution of existing breast tissue)
      1. 0-25%
      2. 25-50%
      3. 50-75%
      4. 75-100%
   3. Volume replacement (>50% of breast tissue excised with implant-based reconstruction and/or locoregional flap reconstruction)
      1. 0-25%
      2. 25-50%
      3. 50-75%
      4. 75-100%
7. Do you perform contralateral balancing procedure?
   1. Yes
   2. No
   3. If no and you are a general surgeon, do you refer this procedure to plastic surgery?
8. If you are a general surgeon, what level do you refer to plastic surgery based on the previous classification system?
   1. Volume displacement level 1
   2. Volume displacement level 2
   3. Volume replacement
9. If you are a general surgeon, is there any other indications, not listed in the classification above that would lead you to refer to a plastic surgeon?
10. Is there any other information you would like to provide regarding the use of oncoplastic surgery in your practice?
11. To what extent do you consider the following as reasons for NOT using oncoplastic breast surgery techniques?

| Reason for not using oncoplastic technique | N/A | Strongly Disagree | Disagree | Agree | Strongly Agree |
| --- | --- | --- | --- | --- | --- |
| I am unfamiliar with these techniques |  |  |  |  |  |
| I am concerned about delay of adjuvant treatment |  |  |  |  |  |
| I am concerned about the need for re-operation for positive margins |  |  |  |  |  |
| I do not have support from plastic surgery |  |  |  |  |  |
| I do not have support from radiation oncology |  |  |  |  |  |
| I am concerned about increased OR time |  |  |  |  |  |
| I am concerned about the lack of specific OHIP billing codes |  |  |  |  |  |
| I am concerned about poor cosmesis |  |  |  |  |  |
| My patients are not interested |  |  |  |  |  |
| I am concerned about the rate of post-operative complications |  |  |  |  |  |
| I am concerned about managing post-operative complications |  |  |  |  |  |
| Other, please specify: |  |  |  |  |  |
